# Schmidt’s Treatment of Parturient Paresis

**Published:** 1900-03

**Authors:** A. G. Alverson

**Affiliations:** Bloomington, Ill.


### REPORTS OF CASES.

### SCHMIDT'S TREATMENT OF PARTURIENT PARESIS.

By A. G. Alverson, V.S.,[1]

BLOOMINGTON, ILL.

Having had an opportunity several times to try the much-vaunted treatment of Schmidt on what he terms parturient paresis, and not being wholly satisfied with my results, thought I would bring the subject before this Society.

Case I.—Grade, shorthorn. Called in the afternoon a short time after the cow had gone down and the second day after eating. My apparatus consisted of a new piece of rubber tubing, a glass funnel, and a milk tube. I thoroughly cleansed the udder of the cow as she lay flat on her side, then disinfected the same. I immersed my apparatus in boiling hot water, made the potassium iodide solution with boiling water, cooled by pouring from one pitcher to another, and forced it into the udder by the law of gravity, thoroughly kneading same. I then rolled the cow upon her sternum and propped her up in that position.

Gave jaborandi (fluid extract) on the tongue; had the rectum thoroughly emptied and left, telling them to keep her in good shape through the night, and I would see her in the morning. They telephoned me the next morning not to come, and I learned later that she died that day. Perhaps further treatment would have been beneficial, but they thought she was getting worse, and so wanted no further expense.

Case II.—Grade, Jersey. In the country; had been down, regained feet, and fell while I was making a general examination. Could not get up, and grew worse rapidly. Gave same treatment per udder, and left fl. ext. jaborandi, to be given later with hypodermatic syringe, if necessary. They gave two doses. This case recovered and was up next day.

Case III.—Grade, shorthorn. Well-marked case complicated with inversion of the vagina up to and including the mouth of the womb, which was easily returned and kept in place. Gave the same treatment as in Case II., but with no success. Saw her again the next day, and repeated the treatment, but that night she succumbed.

[1] Read before the Illinois State Veterinary Medical Association, February 21, 1900.

Case IV. had been down only a short time, but was bad; had treated this cow two years before for the same trouble and gave the same treatment, being minutely careful of every detail and particularly exacting as to asepsis. The following evening had another opportunity to see the animal. She was growing worse, although she had rested quite well all day. Died.

Case V.—This case had recovered under other treatment one year before, and did not appear near as bad this time. Gave the Schmidt in detail, and followed with stimulants, only to lose the patient.

Case VI.—An extra fine cow. Shorthorn and Jersey. Just gone down and several days after calving. Gave full and careful treatment and lost the patient. Am sure I gave as careful detail as possible in the treatment of all these cases, and am inclined to think my poor success was due to the inefficiency of the remedy; still I see that many are claiming great results from it.

That it does exert an influence on the cases I will not question, as most of them, if not all, seemed to brighten up for a time and be far easier to care for and keep in position. Would like very much to be able to do something with it, as it is so much easier to care for the cases in that way.

